# Enhancing targeted strategies for cancer immunotherapy by elucidating mRNA processing mechanisms

**DOI:** 10.3389/fimmu.2026.1844225

**Published:** 2026-05-25

**Authors:** Xiao Lu, Wenwen Gan, Jiang Yuan, Zhihao Chen

**Affiliations:** 1 Geriatric Diseases Institute of Chengdu/Cancer Prevention and Treatment Institute of Chengdu, Department of General Surgery, Chengdu Fifth People’s Hospital(The Second Clinical Medical College, Affiliated Fifth People’s Hospital of Chengdu University of Traditional Chinese Medicine), Chengdu, China; 2 Department of Gastrointestinal Surgery, Sichuan Academy of Medical Sciences & Sichuan Provincial People’s Hospital, University of Electronic Science and Technology of China, Chengdu, China; 3 Department of Thoracic Surgery, Sichuan Medical Science Academy of University of Electronic Science and Technology of China and Sichuan Provincial People’s Hospital, Chengdu, China

**Keywords:** alternative splicing, immunotherapy, mRNA processing, RNA modification, tumor immunity

## Abstract

Immune checkpoint inhibitors have transformed the landscape of cancer therapy; however, the challenge that most patients do not achieve durable benefits urgently necessitates the development of new strategies that extend beyond mere T-cell activation. mRNA processing—comprising alternative splicing, RNA modifications, and RNA editing—establishes a dynamically regulated connection between the intrinsic characteristics of tumors and anti-tumor immunity. This review systematically summarizes how mechanistic insights into these processes can be translated into concrete approaches that enhance the precision of immunotherapy. We first outline how the widely dysregulated splicing events in tumor cells produce abundant neoantigens at a frequency that significantly exceeds that of gene mutations. A subset of these splice isoforms is shared among patients, offering a unique antigen resource for the development of ‘off-the-shelf’ mRNA vaccines, thereby circumventing the manufacturing bottleneck associated with personalized vaccines. Concurrently, RNA modifications driven by N6-methyladenosine (m6A) create an immunosuppressive network at the epitranscriptomics level by bidirectionally modulating the stability of immune checkpoint molecules, e.g., Programmed Death-Ligand 1 (PD-L1), and the functional polarization of macrophages and dendritic cells. In parallel, Adenosine Deaminase Acting on RNA 1 (ADAR1)-mediated Adenosine-to-Inosine (A-to-I) editing designates endogenous double-stranded RNA as ‘self,’ allowing tumors to evade innate immune surveillance and conceal ‘non-self’ signals. This includes the exploitation of splicing-derived neoantigens for designing personalized or shared mRNA vaccines, the deployment of small-molecule inhibitors targeting FTO, Methyltransferase Like 3 (METTL3), and YTH Domain Family Member 2 (YTHDF2) to alleviate immunosuppression, and the utilization of antisense oligonucleotides to precisely modulate splicing factor activity, thereby reversing T-cell exhaustion. Building on this foundation, the combination of these strategies with immune checkpoint blockade has already demonstrated clear synergistic effects in preclinical models and early-phase trials. Additionally, biomarkers based on splicing signatures and expression levels of modification enzymes show promise for accurately stratifying benefiting populations. Despite challenges such as off-target toxicity, intratumoral heterogeneity, and delivery technologies, cutting-edge tools like single-cell and long-read sequencing are rapidly bridging the translational gap. Strategies targeting mRNA processing are advancing cancer immunotherapy from a model of “broad-spectrum activation” to a new paradigm of “precision modulation.”

## 1 Introduction

**Figure 1:**
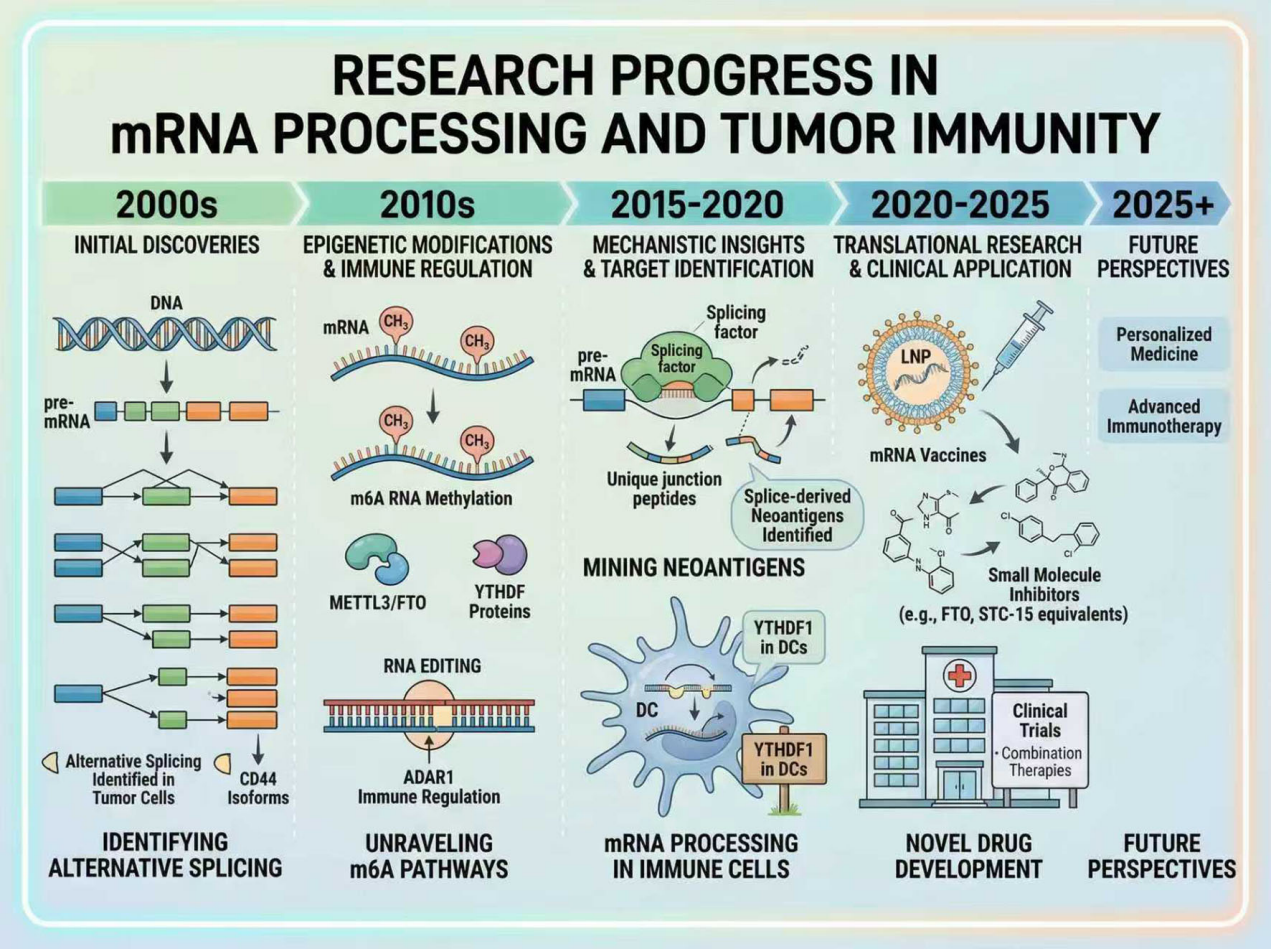
Advances in mRNA processing and tumor immunology research around the year 2000, it was discovered that mRNA in tumor cells can be processed and edited post-transcriptionally to generate novel antigens that are not naturally present in the body. By 2010, research revealed that mRNA methylation in tumor cells is closely associated with tumor initiation and progression. Between 2015 and 2020, studies demonstrated that methylation-mediated processing and modification of mRNA in tumor cells significantly promote tumor progression. From 2020 to 2025, nanoparticle-conjugated mRNA vaccines have been introduced for clinical application in the treatment of cancer patients. This timeline illustrates the evolution of mRNA research in the context of cancer immunotherapy.

The advent of immune checkpoint inhibitors has introduced new therapeutic options for patients with advanced tumors, with anti-Programmed Cell Death Protein 1 (PD-1)/PD-L1 antibodies demonstrating promising clinical efficacy across various solid tumors. However, recent clinical observations indicate that a majority of patients undergoing anti-PD-1 antibody therapy exhibit limited therapeutic responses or develop resistance. This challenge highlights the urgent need for innovative strategies to enhance the efficacy of immunotherapy. Research into the mechanisms of resistance reveals that tumor cells can evade immune recognition through multiple pathways, including functional defects in antigen presentation machinery, such as Major Histocompatibility Complex (MHC) molecules, the creation of an immunosuppressive tumor microenvironment (TME), and T cell exhaustion (1). As a core information carrier of the central dogma, mRNA processing plays a crucial role in the precise regulation of gene expression. A series of post-transcriptional events, including splicing, capping, tailing, base modification, and editing, collectively influence antigen presentation in tumor cells (2). Recently, advancements in high-throughput sequencing and epitranscriptomics technologies have underscored the significant roles of mRNA processing mechanisms in tumorigenesis and immune regulation. Among these, m6A is the most extensively studied epitranscriptomics modification, involved in regulating various aspects of mRNA metabolism (3). Alternative splicing events occur in over 95% of human multi-exon genes, significantly expanding the coding capacity of the genome and closely correlating with the formation of the tumor immunosuppressive microenvironment (4). These studies indicate that mRNA processing constitutes a dynamically regulated complex network that is potentially linked to the efficacy of cancer immunotherapy. This article aims to explore the tumor-specific neoantigens generated by aberrant mRNA splicing and their immunogenic characteristics, the regulatory roles of RNA modifications on immune checkpoint molecules and the immune microenvironment, and the functional significance of RNA editing in self-non-self recognition. It summarizes the mechanisms by which mRNA processing influences the efficacy of cancer immunotherapy, with the goal of augmenting comprehensive treatment strategies for cancer immunotherapy. The timeline of key discoveries in mRNA processing and tumor immunology is presented in (**Figure 1**).

## 2 The core role of mRNA processing mechanisms in tumor immunity

### 2.1 Alternative splicing in tumor cells drives the generation of tumor neoantigens

Alternative splicing, through the selective inclusion or exclusion of exons and introns in precursor mRNA, enables a single gene to produce multiple mRNA transcripts. In comparison to normal tissues, most tumor cells exhibit widespread splicing abnormalities. These aberrant splicing events are hallmarks of cancer and are involved in tumorigenesis and development (5). Pan-cancer analysis further reveals that extensive splicing abnormalities exist in nearly all tumor types, with up to 30% more alternative splicing events in tumors than in normal tissues. On average, each tumor can generate approximately 930 novel splice junctions’ not observed in normal tissues, yielding a vast number of tumor-specific antigens (6). The most significant role of aberrant splicing lies in its potential to generate neoantigens. Many tumor-specific antigens identified in acute myeloid leukemia originate from atypical transcriptional events, such as aberrant alternative splicing (7). As an upstream driver, splicing factor mutations play a critical role in this process. Splicing Factor 3b Subunit 1 (SF3B1), U2 Small Nuclear RNA Auxiliary Factor 1 (U2AF1), Serine/Arginine-Rich Splicing Factor 2 (SRSF2), and other splicing factors are frequently mutated in various hematological malignancies and solid tumors. By disrupting the spliceosome’s precise recognition of splice sites, these mutations lead to global splicing abnormalities (8). Pathways for neoantigen generation from aberrant splicing include intron retention, exon skipping, and activation of cryptic splice sites. These events can cause frameshifts, premature termination, or the creation of novel junctional sequences, leading to the translation of new peptide segments containing tumor-specific amino acid sequences (7). Intron retention typically leads to premature termination codons, triggering nonsense-mediated mRNA decay (NMD), or producing truncated protein isoforms (9). Exon skipping can result in reading frame alterations or functional domain disruption (10).

The resulting splicing-derived neoantigens exhibit high tumor specificity, with some being shared among patients or across various tumor types. In hepatocellular carcinoma, the frequency of aberrant splicing events is over 59 times greater than that of somatic mutations, leading to a population coverage rate of derived antigens reaching 50.94%, which far exceeds the 4.40% coverage of mutation-derived antigens (11). This finding opens the possibility for the development of “universal” immunotherapeutic strategies, such as mRNA vaccines. Once presented on the tumor cell surface by MHC molecules, these neoantigens can be recognized by CD8-positive Cytotoxic T Lymphocyte (CD8+ T cell), thereby triggering an anti-tumor immune response (11). Studies indicate that the predicted Human Leukocyte Antigen (HLA) class I epitope density is significantly lower in peptides encoded by tumor-associated splice variants compared to those derived from normal transcripts, suggesting that aberrant splicing may influence antigen presentation by altering amino acid composition (12).

### 2.2 RNA modification in tumor cells reshapes the tumor immune microenvironment

**Figure 2:**
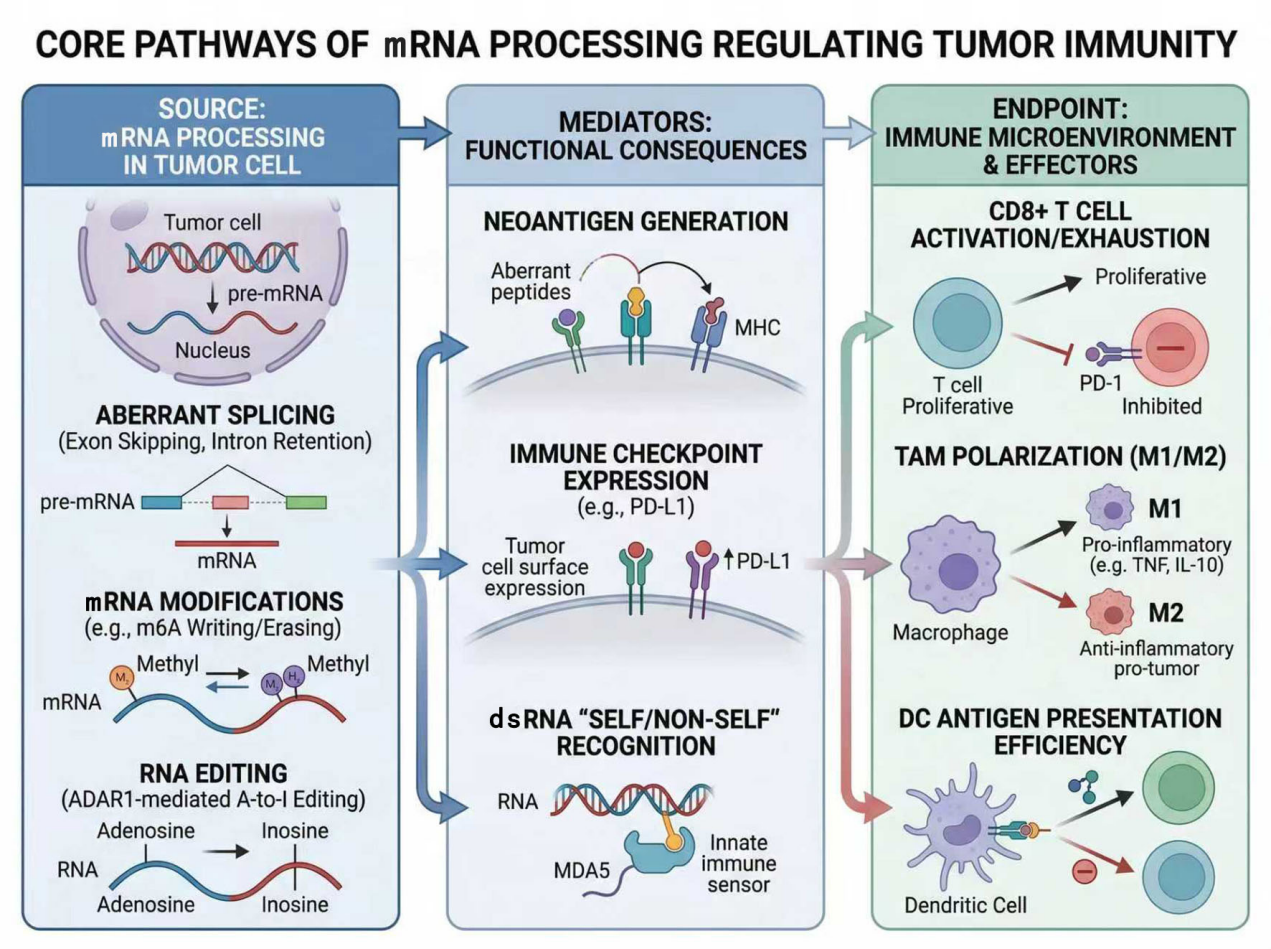
Key pathways regulating mRNA processing in tumor immunity the transcription of tumor cell DNA results in the production of pre-mRNA, which undergoes splicing to yield mRNA that diverges from that of normal host cells. This mRNA is subsequently modified through m6A methylation or A-to-I editing, leading to the generation of tumor-specific antigens. However, while these antigens are presented to T cells via MHC molecules, this process concurrently enhances the expression of PD-L1 on the surface of tumor cells. Moreover, these modifications facilitate the exhaustion of CD8+ T cells and promote the differentiation and proliferation of M2-type macrophages. As a result, this engenders immune tolerance towards tumor cells that express neoantigens, thereby inhibiting the body’s innate and adaptive immune cells from mounting an attack against them.

Key pathways of mRNA processing in tumor immunity are depicted in (**Figure 2**). RNA modifications constitute a highly dynamic regulatory network capable of rapidly responding to microenvironmental signals such as hypoxia and nutrient stress. Their effects are context-dependent, with the same modifying enzyme potentially exerting diametrically opposite effects in different cell types or microenvironmental contexts. Among these, m6A modification is catalyzed by a methyltransferase complex (the “writers”), which includes METTL3, methyltransferase-like 14 (METTL14), and Wilms tumor 1-associated protein (WTAP), dynamically removed by demethylases (the “erasers”), such as fat mass and obesity-associated protein (FTO) and AlkB homolog 5 (ALKBH5), and recognized by binding proteins (the “readers”), such as the YTH domain-containing family (YTHDF) and insulin-like growth factor 2 mRNA-binding protein family (IGF2BP). These processes regulate mRNA metabolism, including stability, splicing, nuclear export, and translation (13). This reversibility makes m6A modification a central hub for the dynamic remodeling of the immune microenvironment. Intrinsic m6A modification in tumor cells directly regulates the expression of immune checkpoint molecules, representing a key pathway for shaping the immunosuppressive microenvironment. In breast cancer, METTL3 enhances PD-L1 mRNA stability in an Insulin-like Growth Factor 2 mRNA-Binding Protein 3 (IGF2BP3)-dependent manner, promoting immune evasion (14). In bladder cancer, the c-Jun N-terminal Kinase (JNK) signaling pathway upregulates METTL3 expression, METTL3 recognizes the m6A modification site on PD-L1 mRNA via Insulin-like Growth Factor 2 mRNA-Binding Protein 1 (IGF2BP1), enhancing its stability and helping tumor cells resist CD8+ T cell-mediated killing (15). In hepatocellular carcinoma, FTO removes m6A modification from FLAD1 mRNA, inhibiting YTHDF2-mediated degradation and upregulating FLAD1 expression, thereby promoting PD-L1 expression and immune evasion (16). In the hypoxic microenvironment of breast cancer, Hypoxia-Inducible Factor 1 Alpha (HIF-1α) transcriptionally activates FTO expression, FTO removes m6A modification from 3-Phosphoinositide-Dependent Protein Kinase 1 (PDK1) mRNA and inhibits YTH Domain Family Member 3 (YTHDF3)-mediated degradation, activating the PDK1/Protein Kinase B (PKB)/Signal Transducer and Activator of Transcription 3 (STAT3) pathway and ultimately upregulating PD-L1 expression (17). This evidence indicates that tumor cells can systematically suppress T cell anti-tumor immunity by regulating PD-L1 expression at multiple levels through the m6A modification network.

At the level of innate immune recognition, ADAR1 marks endogenous double-stranded RNA (dsRNA) as ‘self’ by performing A-to-I editing, thereby preventing its recognition by innate immune sensors such as Melanoma Differentiation-Associated Protein 5 (MDA5) and averting type I interferon-mediated autoimmune activation (18). ADAR1 exhibits a dual regulatory mechanism: one is an editing-dependent manner that modifies dsRNA structure to prevent the activation of pattern recognition receptors, while the other is an editing-independent manner in which it competes with proteins like Protein Kinase R and Z-DNA Binding Protein 1 for dsRNA binding, modulating the intensity of inflammatory signaling. In tumors, ADAR1 not only shields immunostimulatory signals but also regulates tumor cell proliferation, stemness maintenance, and chemotherapy resistance (19). The loss of ADAR1 in glioblastoma can simultaneously trigger an intrinsic stress response in tumor cells and reprogram the immune microenvironment, remodeling it into a pro-inflammatory, anti-tumor state through type I interferon signaling (20). The Epstein-Barr virus EBNA1 protein hijacks ADAR1 to enhance A-to-I editing, thus shielding immunostimulatory dsRNA and remodeling the immunosuppressive microenvironment. Targeting EBNA1 with the Proteolysis Targeting Chimera (PROTAC) degrader EP-1215 can reverse this effect and, when combined with PD-1 antibodies, transform ‘cold’ tumors into a treatment-sensitive phenotype (21). In addition to m6A, 5-methylcytosine (m5C) modification, catalyzed by enzymes such as NSUN2, promotes immune evasion and affects immunotherapy efficacy by regulating immune checkpoint molecules and cytokine networks (22). In summary, RNA epitranscriptomics modifications, represented by m6A and A-to-I editing, form a dynamic regulatory network that tumor cells utilize to systematically reshape the immune microenvironment, thereby facilitating immune evasion.

### 2.3 Impact of mRNA processing in immune cells on immune cell function

mRNA processing mechanisms intrinsically shape the activation, differentiation, polarization, and effector functions of immune cells, encompassing aspects of both alternative splicing and RNA modification.

At the level of alternative splicing, several key nodes in T cell function are subject to splicing regulation. Multiple critical molecules in the T cell receptor signaling pathway undergo splice isoform switching, which affects the strength and threshold of signal transduction (23). During T cell exhaustion, the expression profiles of splice variants of immune checkpoint molecules, such as PD-1 and T-cell Immunoglobulin and Mucin Domain-Containing Protein 3 (TIM-3), are altered, with some splice variants potentially exhibiting distinct functional characteristics (24). Serine/Arginine-Rich Splicing Factor 1 (SRSF1) plays a pivotal role in this intrinsic regulation. The expression of SRSF1 in CD8+ T cells is positively correlated with features of T cell exhaustion and is significantly elevated in CD8+ T cells from patients who do not respond to anti-PD-1 therapy. Inactivation of SRSF1 can enhance glycolytic metabolism and cytotoxic function in CD8+ T cells (25).

At the RNA modification level, m6A modifications and A-to-I editing within immune cells profoundly affect their functional status. In macrophages, m6A modification influences anti-tumor immunity by reprogramming Tumor-Associated Macrophage (TAM) function. The macrophage-specific knockout of Mettl14 leads to the upregulation of Ebi3. EBI3, as a subunit of IL-35, induces functional dysregulation in CD8+ T cells. METTL14-m6A levels are negatively correlated with the levels of dysfunctional T cells (26). YTHDF2, a key reader of m6A modifications, influences macrophage inflammatory cytokine production and antigen presentation capacity by recognizing m6A-modified target transcripts and promoting their degradation. In the hypoxic tumor microenvironment, HIF-1α can directly bind to the promoter region of YTHDF2 to activate its transcription (27). YTHDF2 consolidates the immunosuppressive phenotype of TAMs by degrading pro-inflammatory factor mRNAs and can also promote glycolysis in myeloid-derived suppressor cells (MDSCs) by stabilizing PFKL mRNA, thereby inhibiting CD8+ T cell function (28). YTHDF2 exerts context-dependent immune regulatory effects by degrading or stabilizing diverse transcripts, potentially weakening the cytotoxic function of immune cells or promoting the recruitment of immunosuppressive cells, which shapes an immunosuppressive tumor microenvironment (29). ADAR1 is also involved in regulating macrophage polarization. Its expression level is closely associated with M1/M2 polarization status; high ADAR1 expression promotes M2-type polarization through the ADAR1-miR-21-Foxo1-IL-10 axis. Knocking down ADAR1 can enhance the inflammatory response of macrophages and promote M1 polarization (30). In colon cancer, cancer cells transport ADAR1 protein to macrophages via exosomes, promoting RNA editing of AZIN1 and GLI1, which induces TAM polarization towards the M2 phenotype that secretes SPP1 to activate the Nuclear Factor Kappa B pathway in cancer cells, leading to drug resistance (31).

The m6A modification in dendritic cells (DCs) plays a crucial role in regulating anti-tumor immunity. YTH Domain Family Member 1 (YTHDF1), which is highly expressed in DCs, enhances antigen degradation by promoting the translation of lysosomal protease-related transcripts, thereby impairing the efficiency of cross-presentation. The deletion of YTHDF1 significantly enhances the ability of DCs to cross-present antigens and boosts CD8+ T cell anti-tumor immunity (32). Additionally, METTL3 regulates the translation of co-stimulatory molecules such as Cluster of Differentiation 40 (CD40) and Cluster of Differentiation 80 (CD80) through m6A modification; specifically, the DC-specific knockout of METTL3 impairs T cell activation function (33). Consequently, the dual regulation of antigen presentation and co-stimulatory signals by the m6A modification network in DCs is critical in determining the intensity of T cell activation, serving as an important control point for the immune status of the microenvironment. In T cells, ADAR1 regulates T cell homeostasis, development, and peripheral function through both editing-dependent and editing-independent mechanisms (34). Following T cell receptor activation, ADAR1-mediated A-to-I editing is significantly upregulated. T cell activation stimuli can induce an approximate fourfold increase in the expression of ADAR1 isoforms and introduce site-selective editing in transcripts related to the T Cell Receptor (TCR) signaling pathway. This suggests that ADAR1 may fine-tune the gene expression network during T cell activation through RNA editing (35).

## 3 Immunotherapeutic strategies based on mRNA processing

### 3.1 Mining the splicing-derived neoantigen repertoire: from personalized vaccines to “off-the-shelf” shared targets

Splicing-derived neoantigens possess unique advantages, with development strategies evolving from personalized customization towards ‘off-the-shelf’ shared targets. First, splicing abnormalities are extremely common in tumors, occurring far more frequently than gene mutations; approximately 2%-3% of splicing alterations have the potential to generate immunogenic peptides recognizable by T cell receptors (36). Second, splice variants often exhibit tumor-type specificity or even patient specificity, enabling truly personalized targeting (37). Third, some splice variants are shared among multiple patients and can be developed as ‘off-the-shelf’ immunotherapy targets. Recent research has identified 789 tumor-specific shared splicing events across 10 cancer types, among which splicing-derived neoantigens originating from RPL22 and GNAS can be specifically recognized by CD8+ T cells, inducing tumor-killing effects. These antigens are characterized by high intra-tumoral conservation (38). The shared neoantigens originate from recurrent splicing patterns driven by dysregulation of specific splicing factor expression. Compared to personalized neoantigen vaccines, they offer advantages in large-scale manufacturing and quality control, potentially significantly reducing manufacturing costs and treatment cycles (39).

From a technical pathway perspective, the discovery of splicing-derived neoantigens relies on the integrated analysis of RNA sequencing (RNA-seq) and immunopeptidomics. RNA-seq can identify aberrantly expressed splice variants in tumor tissues. Computational pipelines, such as SNAF, systematically predict splicing-derived neoantigens, while immunopeptidomics, combined with mass spectrometry, can confirm whether peptides derived from these variants are genuinely presented by MHC molecules on the tumor cell surface (40). The latest proteomics platforms have enabled the direct identification of neoantigen peptides from formalin-fixed paraffin-embedded (FFPE) tumor tissues. This not only validates predicted classical neoantigens but also uncovers non-canonical peptides, which may originate from non-coding open reading frames, proteasomal splicing abnormalities, or RNA splicing abnormalities, and cannot be traced back to the genomic source (41).

At the application level, personalized mRNA vaccines have recently achieved significant clinical breakthroughs. In the context of pancreatic cancer, the personalized mRNA-lipid complex vaccine autogene cevumeran (also known as BNT122) has been shown to induce durable, tissue-resident CD8+ T cells and prolong recurrence-free survival (42). Concurrently, actively intervening in the RNA splicing process to generate endogenous tumor neoantigens presents a novel strategy for enhancing tumor immunogenicity. Pharmacological modulation of RNA splicing, such as the use of Indisulam (E7070) to degrade RNA Binding Motif Protein 39 (RBM39) or MS-023 to inhibit type I PRMT enzymes, can yield bona fide splicing-derived neoantigens, stimulate T cell-dependent anti-tumor immunity, and exert synergistic effects with immune checkpoint blockade (43). Building upon this foundation, researchers have further encapsulated BaTiO_3_ nanoparticles and the splicing modulator Indisulam within a PD1-overexpressing cell membrane shell, achieving tumor targeting and ultrasound-triggered, controlled release of components. This innovative strategy generates high-quality endogenous tumor neoantigens by interfering with RNA alternative splicing (44). In addition to directly modulating the spliceosome, inhibiting the NMD pathway represents another strategy to enhance the presentation of splicing-derived neoantigens. NMD is a conserved post-transcriptional quality control mechanism in eukaryotes that maintains transcriptome integrity and regulates gene expression by targeting mRNAs containing premature termination codons (45). Inhibiting key NMD factors such as UPF1 or SMG1 stabilizes these transcripts and promotes the translation of non-canonical proteins, thereby increasing the repertoire of neoantigen peptides presented by MHC molecules (46). Furthermore, the development of ‘off-the-shelf’ vaccines based on shared splicing neoantigens is accelerating, promising to further expand the patient population that can benefit from these advancements.

### 3.2 Targeting RNA modification enzymes: reversing immunosuppression and enhancing anti-tumor immunity

Targeting RNA modification enzymes aims to reverse immunosuppression and enhance anti-tumor immunity by interfering with m6A writers, erasers, or readers. The key lies in achieving selectivity within the therapeutic window. FTO, an m6A demethylase, is overexpressed in various tumors and facilitates immune evasion. Inhibition of FTO can downregulate glycolytic genes, reduce lactate production, and restore dendritic cell maturation and T cell function (47). This mechanism involves the regulation of m6A demethylation of transcription factors such as c-Jun and JunB (48). The small-molecule FTO inhibitor Dac51 can block immune evasion and produce synergistic effects with anti-PD-L1 therapy (49). Another FTO inhibitor, Dac590, developed through structure-guided design, significantly inhibited tumor growth and prolonged survival in an acute myeloid leukemia mouse model upon oral administration, with no observed toxicity (50).

METTL3, the core catalytic subunit of the m6A methyltransferase, represents another key target. The METTL3 inhibitor STC-15 suppresses tumor growth through direct anti-tumor effects and anti-cancer immune responses (51). It has achieved a 9% objective response rate and a 67% disease control rate in advanced solid tumors and has entered Phase Ib/II clinical trials in combination with the PD-1 antibody toripalimab (52). METTL14 exhibits unique effects in specific tumors; its knockdown can enhance CD8+ T cell activation (53).

Strategies targeting RNA modification readers are emerging as significant avenues in cancer research. YTHDF2 plays a pivotal role at the intersection of tumor immunity and oncogenic signaling pathways (54). Small molecule inhibitors that target YTHDF2 have the potential to reverse immunosuppression and enhance the efficacy of immune checkpoint blockade (55). Furthermore, drugs aimed at other RNA modification enzymes, such as METTL1, ADAR1, and NAT10, are currently in preclinical development, positioning these pathways as promising new therapeutic targets, particularly for hematologic malignancies (56). Overall, FTO and METTL3 inhibitors represent relatively advanced targets, having progressed into clinical trials. In contrast, strategies targeting YTHDF2, NSUN2, and ADAR1 remain in preclinical or early development stages, necessitating careful consideration of their potential interference with normal immune cell functions during clinical translation.

### 3.3 Modulating splicing factors: reversing T cell exhaustion and restoring effector function

T cell exhaustion represents a fundamental mechanism underlying resistance to immunotherapy, with splicing factors serving as critical regulators of T cell exhaustion states. CWF19 Like Cell Cycle Control Factor 1 (CWF19L1), a newly identified splicing regulatory factor, positively influences T cell effector function; its deletion results in the downregulation of cytotoxic molecule expression, thereby impairing the anti-tumor efficacy of CD8+ T cells (57). SRSF1 exhibits dual regulatory characteristics, as its inactivation can enhance glycolytic metabolism and cytotoxic function in CD8+ T cells, thereby improving the efficacy of adoptive T cell therapy. Conversely, SRSF1 also suppresses glycolytic metabolism in tumor cells, thereby removing the metabolic barriers to T cell activation. This dual functionality makes SRSF1 a unique target for enhancing anti-tumor immunity from both immune cell-intrinsic and tumor cell-extrinsic perspectives (25).

In the context of Regulatory T Cells (Tregs), the Forkhead Box P3 (FOXP3) splice isoforms FOXP3-FL and FOXP3-ΔE2 demonstrate significant differences in their suppressive functions (58). Preclinical studies have confirmed that mice expressing only FOXP3ΔE2 exhibit resistance to multiple tumors, characterized by reduced intratumoral Treg suppressive activity and enhanced CD8+ T cell activation. Moreover, a morpholino oligonucleotide designed to induce FOXP3 exon 2 skipping has been shown to enhance anti-tumor immunity in mouse models and patient-derived tumor organoids (59). The primary advantage of this strategy lies in its ability to reduce Treg suppressive function through splicing modulation, rather than through systemic Treg depletion, which may potentially mitigate autoimmune-related adverse events.

At the tool level for targeting splicing regulation, antisense oligonucleotides (ASOs) can precisely modulate splicing patterns. An ASO targeting PD-1 exon 3 induces the production of soluble PD-1 by blocking the binding of Serine/Arginine-Rich Splicing Factor 3 (SRSF3) to an exonic splicing enhancer, thereby enhancing anti-tumor immunity (60). Unlike antibodies that directly block PD-1/PD-L1 binding, this strategy attenuates the inhibitory signal of membrane-bound PD-1 at the post-transcriptional level by altering its splicing pattern, offering a differentiated approach to reversing T cell exhaustion. Collectively, these strategies point towards a core goal—restoring the anti-tumor effector function of T cells through precise modulation of splicing events.

## 4 Clinical application prospects and challenges of mRNA processing-modifying drugs in tumor therapy

### 4.1 Combining mRNA processing-targeted therapies to improve tumor immunotherapy response

**Figure 3:**
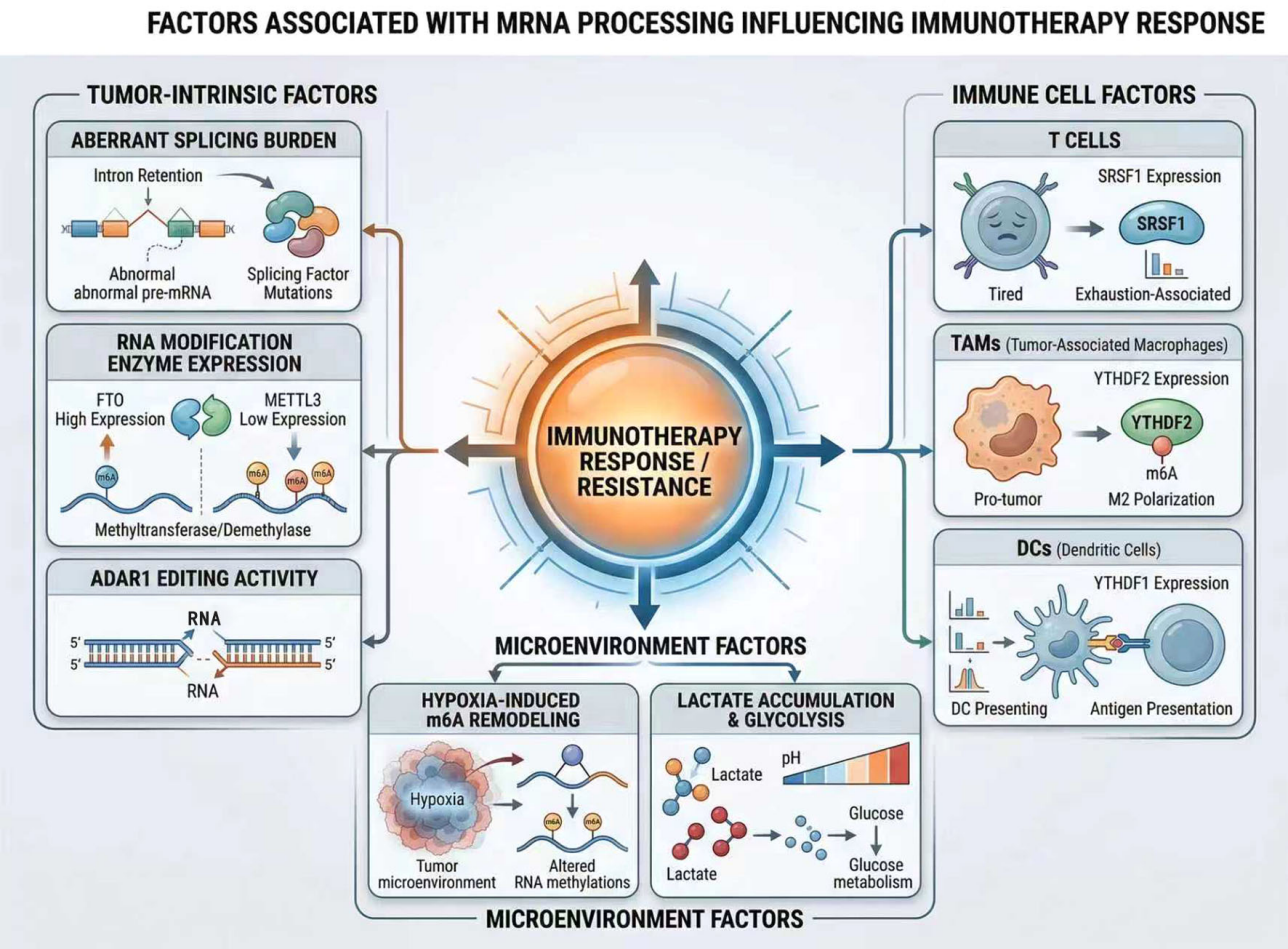
Factors influencing mRNA processing and their impact on immunotherapy responses three key factors influence the efficacy of mRNA therapeutics when combined with immunotherapy. First, the mRNA processing status of tumor cells, which includes abnormal splicing of pre-mRNA, the activity of RNA-modifying enzymes, and the enzymatic activity of ADAR1. Second, the state of the tumor immune microenvironment, characterized by local hypoxia, energy and nutrient deprivation, and lactate accumulation. Third, the impact of mRNA processing on immune cell function. For instance, SRSF1 is associated with CD8+ T cell exhaustion, YTHDF2 correlates with macrophage polarization, and YTHDF1 is linked to the antigen-presenting capacity of dendritic cells.

The key factors affecting mRNA processing and immunotherapy responses are summarized in (**Figure 3**). mRNA processing-targeted therapies can enhance the responses of tumor immunotherapy through various combination strategies. The rationale for synergistically combining mRNA processing-targeted therapies with immune checkpoint blockers (ICBs) is rooted in their dual complementarity. The former reduces the tumor’s capacity for immune evasion and ameliorates the suppressive state of the microenvironment by intervening in splicing or RNA modifications. The latter blocks the PD-1/PD-L1 signaling axis, thereby releasing the function of effector T cells. This creates a synergistic loop characterized by ‘relieving suppression and activating the effector.

Regarding m6A modification enzymes, the synergistic effects of FTO inhibitors in conjunction with ICBs have been validated across multiple models. The FTO inhibitor CS2 enhances the activation and recruitment of CD8+ T cells by upregulating the m6A modification level of GPNMB and promoting YTHDF2-mediated degradation, thereby producing synergistic effects with anti-PD-1 therapy and sorafenib (61). In melanoma, FTO siRNA combined with sonodynamic therapy remodels the immune microenvironment and synergizes effectively with anti-PD-L1 therapy (47). The METTL3 inhibitor STC-15 is currently undergoing Phase Ib/II clinical trials in combination with the anti-PD-1 antibody toripalimab for the treatment of advanced solid tumors (52). Furthermore, METTL3 drives M2 polarization in TAMs in esophageal cancer through m6A modification of CXCR4, indicating that combinations with ICBs may offer significant therapeutic breakthroughs (62).

In the field of mRNA vaccines, the combination of splicing-derived neoantigen vaccines with ICBs has entered the clinical validation phase. Five-year follow-up data from the Phase IIb trial of the personalized neoantigen mRNA vaccine, mRNA-4157, combined with pembrolizumab, demonstrated a 49% reduction in the risk of recurrence or death in the combination group (Hazard Ratio=0.510) (63). If “off-the-shelf” vaccines based on shared splicing neoantigens can be combined with ICBs, they are expected to further expand the population of beneficiaries. At the technology platform level, lipid nanoparticles co-delivering an mRNA vaccine with a cyclic GMP-AMP synthase (cGAS) agonist can enhance antigen presentation and CD8+ T cell activation by inducing type I interferon, significantly improving efficacy when combined with ICBs (64). Furthermore, nanosystems loaded with mRNA and photothermal agents have achieved local immune remodeling and enhanced CD8+ T cell infiltration in a breast cancer model (65). Targeted modification of lipid nanoparticles can enable selective delivery of mRNA vaccines (66), and the co-delivery of mRNA vaccines with immune agonists can further enhance anti-tumor immune responses (67). The integration of Clustered Regularly Interspaced Short Palindromic Repeats (CRISPR) technology with mRNA vaccines is emerging as a new direction in precision immunotherapy; sequential CRISPR editing followed by personalized mRNA vaccination strategies has demonstrated technical feasibility in early clinical trials (68).

Biomarkers derived from mRNA processing characteristics provide a novel approach for identifying patient populations that are likely to benefit from therapies targeting mRNA processing in combination with ICBs. By analyzing RNA-seq data from tumor tissues, quantitative indicators can be extracted to reflect the extent of aberrant splicing. These indicators include the total number of aberrant splicing events, the burden of splicing factor mutations, and the expression levels of specific splice variants (69). Bioinformatics tools, such as SpliceMutr, systematically identify tumor-specific splicing-derived neoantigens and calculate a splicing antigenicity score. This score has been positively correlated with treatment response in melanoma immunotherapy cohorts (70), representing a biomarker supported by retrospective clinical validation data. The next step is to confirm its predictive power in prospective cohorts. In lung cancer, long-read RNA sequencing has revealed that an intron retention event in the Signal Transducer and Activator of Transcription 2 (STAT2) gene effectively distinguishes responders from non-responders to anti-PD-L1 therapy (71), also representing a biomarker with existing clinical utility evidence. In breast cancer, the Epithelial Splicing Regulatory Protein 1 (ESRP1)-driven splicing event burden significantly correlates with T cell expansion following ICB and can predict long-term survival benefits (72). However, its predictive threshold and applicable cancer types have not been standardized, classifying it as a biomarker with initial potential but still lacking independent validation. At the tumor biology level, CD44 splice isoform switching regulated by ESRP1 affects immune cell infiltration patterns (73). Associations between intron retention levels and T cell infiltration characteristics have also been reported in clear cell renal cell carcinoma (74). These findings suggest that specific patterns of splicing events might reflect the immune microenvironment status of tumors, but they have not been directly linked to ICB efficacy, classifying them as candidate biomarkers still in the discovery phase.

At the level of RNA modification enzymes, FTO is overexpressed in various tumors. Patients with high FTO expression may exhibit increased sensitivity to FTO inhibitors when combined with ICB regimens (75). In breast cancer, individuals with elevated FTO expression are more likely to benefit from FTO inhibitors in conjunction with PD-1/PD-L1 blockade (17). In gastric cancer, the expression level of ALKBH5 correlates with the response to anti-PD-1 immunotherapy (76). Additionally, ADAR1 expression is associated with resistance to combination therapy; aberrant ADAR1 expression in tumors maintains immune tolerance by inhibiting the double-stranded RNA sensing pathway, potentially diminishing the efficacy of ADAR1 inhibitors combined with ICB (77). Conversely, certain tumors that rely on ADAR1 for immune surveillance evasion may exhibit increased sensitivity to this combination regimen (78). Furthermore, the expression of the splicing factor U2SURP in melanoma is negatively correlated with immune infiltration scores, as well as the infiltration levels of DCs and natural killer (NK) cells (79). The aforementioned RNA modification enzyme and splicing factor biomarkers are currently at a stage where they exhibit preliminary predictive potential; however, they lack independent validation. Their predictive thresholds, applicable cancer types, and detection methods are not yet standardized across the field, and independent cohort replication is absent. The transition from discovery to application encounters significant bottlenecks in this area. The establishment of a composite biomarker system that integrates splicing profiles, modification enzyme expression profiles, and immune infiltration characteristics remains necessary. The expression of RNA modification enzymes is dynamically regulated by microenvironmental signals, which complicates the ability of a single-time-point test to accurately reflect adaptive changes during treatment. Furthermore, the lack of standardization in detection methods and interpretation criteria hinders translation into routine clinical testing. Overcoming these bottlenecks is a critical step in advancing from ‘biomarker description’ to ‘precision patient stratification.

### 4.2 Challenges of mRNA processing-modifying drugs in clinical tumor therapy

While strategies targeting mRNA processing mechanisms demonstrate considerable potential, they encounter several fundamental challenges during clinical translation. Notably, off-target toxicity, tumor heterogeneity, and limitations in mechanistic research represent significant unresolved issues. Off-target toxicity poses the most pressing safety concern. mRNA processing mechanisms—including splicing, modification, and editing—are equally vital in normal cells, particularly for maintaining the homeostasis of immune cells (80). Global regulation of m6A modification or splicing factor activity may disrupt the normal development and effector functions of immune cells, resulting in a narrow therapeutic window. A core advantage of targeting m6A modification enzymes lies in their dynamic reversibility; writers, erasers, and readers together form a bi-directionally regulatable network, theoretically enabling more precise interventions than gene editing. However, this same advantage poses a significant barrier to clinical translation. m6A modification is essential for the development, differentiation, and effector functions of normal immune cells, and systemic inhibition of METTL3 or FTO could potentially disrupt key immune processes such as T cell activation and dendritic cell maturation, thereby leading to a narrow therapeutic window. For instance, systemic knockout of ADAR1 can result in type I interferonopathy and early mortality. Mice with liver-specific ADAR1 knockout exhibit growth retardation, high mortality, and severe liver inflammation, underscoring the need for careful balancing between efficacy and safety in drug development targeting this pathway (81). Similarly, splicing factors are widely expressed in various normal tissues, and their systemic inhibition could produce unintended toxic side effects (80). The selectivity of current small molecule inhibitors remains suboptimal; thus, finding methods to inhibit target activity in tumors without affecting the same target in normal tissues is an area urgently requiring breakthroughs in medicinal chemistry and delivery technologies.

Intra-tumoral heterogeneity presents a significant challenge in cancer treatment. mRNA processing events can vary considerably among different regions within a tumor and across distinct clonal subpopulations. Single-cell splicing sequencing has demonstrated that certain splice variants are expressed exclusively in rare subpopulations, which may serve as a critical source of drug-resistant relapse (82). This finding has profound implications for the development of targeted therapies. Interventions aimed at a single splicing event or enzyme modification may only eliminate a subset of tumor clones, while the surviving subpopulations expand under therapeutic pressure, ultimately leading to immune evasion and treatment resistance. Furthermore, tumors can evade immune recognition through frequency-dependent selection of neoantigens; high-frequency neoantigen clones are eliminated, allowing rare clones to survive and proliferate (83). This dynamic evolutionary process necessitates that immunotherapy strategies possess the capability to cope with antigen loss and clonal selection, imposing higher demands on the design of splicing-derived neoantigen vaccines.

At the level of fundamental mechanisms, most studies remain at the stage of correlational description, lacking in-depth validation of functional causality. The dynamic changes in mRNA processing events in response to tumor microenvironment signals, as well as the topological structure and key nodes of this regulatory network, remain unclear. Research indicates that m6A methylation is involved in the metabolic reprogramming of tumor cells and the phenotypic switching of immune cells under hypoxic conditions, which synergistically promotes the formation of an immunosuppressive microenvironment. However, the specific regulatory network remains to be elucidated (84). In terms of translational application, the clinical development of splicing-derived neoantigens faces multiple logistical bottlenecks. Current neoantigen prediction algorithms encounter limitations in accurately predicting immunogenicity. The actual immunogenicity of most predicted peptides awaits functional validation. Additionally, the long personalized preparation cycle and high production costs pose practical obstacles that limit their widespread application (85). Concurrently, the screening and validation of biomarkers lack support from large-scale prospective clinical trials, and their predictive performance and clinical utility require further confirmation (86). These translational obstacles, intertwined with limitations in understanding fundamental mechanisms, constitute significant bottlenecks from mechanistic discovery to clinical intervention.

Faced with these challenges, cutting-edge technologies are providing new pathways to address these issues. Advances in single-cell splicing sequencing technology enable researchers to elucidate the splicing landscapes of tumor and immune cells at single-cell resolution, directly tackling the challenge of tumor heterogeneity. Traditional bulk RNA sequencing often masks characteristic splicing events in rare subpopulations, whereas single-cell splicing sequencing can uncover these subpopulations and their relationship with treatment resistance (72). Long-read sequencing technologies, particularly nanopore sequencing, can directly detect RNA modifications without the need for antibody enrichment or chemical treatment, thus facilitating the dynamic monitoring of modifications such as m6A and m5C. This technology has been successfully utilized to map RNA modification profiles in clinical tumor samples, holding promise as a companion diagnostic tool to guide immunotherapy selection (87). More importantly, long-read sequencing can directly capture full-length splice isoforms, overcoming the limitations of short-read sequencing in identifying splice variants, thereby providing technical support for the discovery of novel splicing-derived neoantigens (88). In the realm of multimodal combination therapy, mRNA-based Chimeric Antigen Receptor (CAR) cell manufacturing technology can mitigate the genomic integration risks associated with viral vectors, achieving transient and controllable CAR expression, thus offering a novel tool for localized treatment of solid tumors. The integrated application of these cutting-edge technologies will advance precision immunotherapy based on mRNA processing mechanisms from concept to clinical reality.

## 5 Conclusion: a new paradigm moving from “broad-spectrum immune activation” to “precision-targeted regulation”

mRNA processing mechanisms—alternative splicing, RNA modifications, and RNA editing—collectively serve as a critical bridge connecting the intrinsic characteristics of tumor cells to the extrinsic functions of immune cells. At the tumor cell level, aberrant splicing drives neoantigen generation through pathways such as intron retention and exon skipping, occurring at a frequency that far exceeds that of somatic mutations. Some of these neoantigens are shared among patients, providing a population-level resource base for ‘off-the-shelf’ vaccines. RNA modifications, represented by m6A and A-to-I editing, reshape the suppressive immune microenvironment through two dimensions: immune checkpoint activation and innate immune evasion—by upregulating PD-L1 expression and shielding the immunogenicity of endogenous dsRNA. At the immune cell level, alternative splicing and RNA modifications intrinsically determine T cell exhaustion, Treg suppressive function, and macrophage polarization direction, constituting a regulatory dimension that cannot be overlooked in immunotherapy.

Cancer immunotherapy is undergoing a paradigm shift from ‘broad-spectrum immune activation’ to ‘precision-targeted regulation.’ Target selection is transitioning from pan-immune signal molecules to specific splicing factors, modifying enzymes, or splicing-derived neoantigens. Therapeutic strategies are evolving from single ICB to diverse combinations involving splicing regulation, modification enzyme inhibition, and mRNA vaccines. Patient screening is progressing from empirical drug administration to biomarker-based stratification utilizing splicing signature profiles and modification enzyme expression profiles.

In terms of clinical maturity, various strategies exhibit a distinct gradient. Inhibitors of FTO and METTL3 have progressed to clinical trials, and Phase IIb data for splicing neoantigen vaccines in combination with ICB have demonstrated significant efficacy signals, marking these as the most advanced strategies approaching clinical application. Conversely, targets such as YTHDF2 and ADAR1, along with strategies focused on splicing factor modulation to reverse T cell exhaustion, remain in preclinical or early development stages, categorizing them as exploratory targets. Additionally, biomarker systems utilizing single-cell and long-read sequencing are currently in the phases of technological accumulation and validation. This gradient distribution indicates that clinical translation should implement a tiered advancement strategy, which accelerates the mechanistic validation of exploratory targets while prioritizing clinical trials for more mature targets. A summary of therapeutic strategies and clinical development stages is provided in (**Table 1**).

**Table 1:** Summary of therapeutic strategies targeting mRNA processing mechanisms and their clinical development stages.

Therapeutic target/strategy	Mechanism of action	Representative indications	Clinical development stage	Key references
FTO Inhibitors	Inhibit the m6A demethylase FTO, upregulate PD−L1 mRNA degradation, and restore T cell function	Liver cancer, Melanoma, Acute Myeloid Leukemia	Clinical trial stage	(48)、 (50)
METTL3 Inhibitors	Inhibit the m6A methyltransferase METTL3, reduce PD−L1 stability, and relieve immunosuppression	Advanced solid tumors, Bladder cancer	Clinical trial stage	(15)、 (52)
Personalized mRNA Neoantigen Vaccines	Encode patient−specific splicing/mutation neoantigens to induce T cell responses	Pancreatic cancer, Melanoma	Clinical trial stage	(42)、 (63)
Shared Splicing Neoantigen “Off−the−Shelf” Vaccines	Target public neoantigens generated by recurrent aberrant splicing shared among patients	Glioma and various solid tumors	Discovery/Preclinical	(38)、 (40)
YTHDF2 Inhibitors	Inhibit the m6A reader YTHDF2 and reverse the immunosuppressive tumor microenvironment	Various solid tumors	Preclinical/Early development	(29)、 (55)
Splicing Factor Modulation (SRSF1/FOXP3/PD−1)	Enhance CD8^+^ T cell function, reduce Treg suppressive activity, or induce soluble PD−1 production	Various solid tumors	Discovery/Preclinical	(25)、 (59)
Pharmacological Splicing Modulation (RBM39 Degradation)	Induce global splicing abnormalities, generating endogenous neoantigens	Various solid tumors	Discovery/Preclinical	(43)
NMD Pathway Inhibition	Stabilize aberrant transcripts containing premature termination codons, increasing neoantigen presentation	Various solid tumors	Discovery/Preclinical	(45)、 (46)
ADAR1 Inhibitors	Relieve ADAR1−mediated editing shielding of self−dsRNA, activating type I interferon responses	Glioblastoma, Breast Cancer Susceptibility Gene−mutant tumors	Discovery/Preclinical	(20)、 (78)

To actualize the vision from concept to practice, core challenges such as off-target toxicity, tumor heterogeneity, and limitations in mechanistic understanding must be thoroughly addressed. The role of mRNA processing is equally essential in normal immune cells, and the heterogeneous distribution and clonal evolution of splicing events within tumors necessitate therapeutic strategies that possess dynamic response capabilities. Looking ahead, the integrated application of cutting-edge technologies, including single-cell multi-omics, long-read sequencing, and next-generation RNA therapeutics, is anticipated to advance precision immunotherapy based on mRNA processing mechanisms from concept to clinical practice, thereby providing more durable and personalized benefits to cancer patients.
